# Effects of air pollution on global health: evidence from the global burden of disease study in the BRICS countries

**DOI:** 10.1007/s00420-024-02087-7

**Published:** 2024-07-12

**Authors:** Deepak Kumar Behera, Pozhamkandath Karthiayani Viswanathan, Sanghamitra Mishra

**Affiliations:** 1 Department of Economics and Finance, The Business School, RMIT International University Vietnam, Ho Chi Minh City, 700000 Vietnam; 2 Amrita School of Business Amritapuri, Amrita Vishwa Vidyapeetham, Kollam, Kerala 690 525 India; 3 School of Public Health, AIPH University, Bhubaneswar, Odisha 751002, India

**Keywords:** Air pollution, Global Burden of Disease, Health effects, BRICS, India, NCDs

## Abstract

**Purpose:**

Considering the dynamic influence of environmental, social, economic, and political factors in the emergence and growth of the BRICS countries (Brazil, Russia, India, China, and South Africa) over the years and pre-existing differences, the adverse effects of air pollution on the health and well-being of the people have remained major areas of academic inquiry and policy interventions. The present study examines the global trend of deaths and Disability Adjusted Life Years (DALYs) attributable to air pollution with particular reference to the BRICS countries for the period 1990 to 2019.

**Methods:**

This study has used the global burden of disease estimates by using different rounds of the Global Burden of Disease (GBD) study report published by the Institute of Health Metrics Evaluation. This study has calculated the cause of death and DALYs due to environmental risk factors (i.e. Air pollution). Data analysis has been done by using the standard formula for the calculation of death (mortality) rate and DALYs rate. Similarly, we calculated the age and gender-wise death and DALYs rate by using the appropriate numerator and denominator.

**Results:**

The study discovered a significant shift in disease patterns over this period, as communicable diseases like respiratory infections and tuberculosis were replaced by non-communicable diseases such as ischemic heart disease (17.2 million), chronic obstructive pulmonary disease (14.59 million), and stroke (17.02 million) as the primary causes of air pollution-related deaths in 2019 at the global level. Additionally, the study identified a worrying increase in deaths linked to neonatal disorders and respiratory infections caused by ambient particulate matter pollution in South Africa, India, and Brazil. The impact of air pollution on public health is evident across different age groups and genders, with people aged 50-69 years, those aged 70 and above, and children under 5 years being more vulnerable. Furthermore, the male population is disproportionately affected by communicable and noncommunicable diseases caused by air pollution.

**Conclusion:**

The study highlights the need for policymakers to implement evidence-based interventions to tackle this global health problem. The interventions should aim to reduce the emerging crisis of non-communicable diseases related to air pollution, particularly among vulnerable age groups and the male population, ultimately improving public health outcomes.

## Introduction

Researchers and practitioners have been studying the exposure–response characteristics of air pollution and its impact on various health conditions for several decades now. The general public is also aware of the adverse effects of air pollution on health and the environment. Studies on urban air pollution have revealed that urbanization has a significant impact on the level of air pollution and its negative effects on the health and well-being of the urban population (Liang et al. 2019; Mabahwi et al. 2014). A study conducted on air pollution in different Latin American countries revealed that the most polluted cities are not necessarily the capital cities, indicating the widespread nature of poor air quality in several cities (Jorquera et al. 2019).

Indoor air pollution from solid fuels remains one of the biggest threats to people's lives across the world, particularly in low and middle-income countries (Rentschler, and Leonova 2023; Gkidou et al. 2017). Solid fuel combustion is one of the top ten environmental risks for the global burden of disease (GBD), leading to a significant number of premature deaths and disabilities (Zhang and Smith 2003). This problem persists in developing countries due to heavy reliance on coal, biomass, and stoves with incomplete combustion (Bruce et al. 2000; Pandey et al. 2021).

Ambient particulate matter air pollution has witnessed a notable global increase, as highlighted by Shaddick et al. (2020). This rise can be attributed to various sources such as traffic, domestic wood combustion heaters, industrial activities, coal-powered stations, natural dust, salt, diesel exhaust, and others, as indicated by Hime et al. (2018) and Karagulian et al. (2015). Despite efforts to reduce particulate matter levels over time, their adverse effects on human health remain significant. Ambient ozone air pollution stands out as a significant contributor to air quality concerns, with prolonged exposure linked to respiratory conditions, cardiovascular diseases, and metabolic disorders (Katsouyanni 2003; Nuvolone et al. 2018). While instances of short-term exposure to ozone and its direct impact on human health are relatively uncommon, the established association cannot be ignored. However, it's worth noting that studies such as Zhao et al. (2017) present contrasting evidence. Their systematic review and meta-analysis shed light on the potential connection between short-term exposure to air pollutants, particularly ozone, and the occurrence of out-of-hospital cardiac arrest, suggesting a need for further investigation and nuanced understanding.

The detrimental impacts of air pollution extend globally, but their distribution is far from uniform, even within BRICS nations—Brazil, Russia, India, China, and South Africa—despite their shared economic and demographic traits (Tan et al. 2021). This divergence implies differing levels of exposure and vulnerability to air pollution-related health hazards among various regions and demographics within BRICS countries. Recognizing these discrepancies is imperative for crafting tailored interventions and policies to address the adverse effects of air pollution on both public health and environmental sustainability. While air pollution's adverse effects are widespread, they manifest unevenly, even within BRICS countries, characterized by economic and demographic parallels (Liu et al. 2022; Behera and Karthiayani 2022; Vedal 1997). This disparity underscores variations in exposure levels and susceptibility to air pollution-related health risks across regions and populations within BRICS nations. Grasping these distinctions is essential for designing targeted interventions and policies to mitigate the detrimental impacts of air pollution on public health and environmental well-being.

BRICS countries are considered the most emerging economies in the world but also have a significant burden of diseases (Marinho et al. 2018). Healthcare burden associated with infectious diseases, such as respiratory infections, tuberculosis, HIV/AIDS, STIs, enteric diseases, and malaria, is proportionately high among the BRICS countries out of the total global infectious disease burden (Liu et al. 2022). The recent SARS COVID-19 pandemic had a significant impact on the BRICS countries, with more than a quarter of the total cases worldwide (Zhu et al. 2021). In 2019, BRICS countries accounted for more than 60% of total deaths associated with chronic respiratory diseases (Bai et al. 2022). The fastest-growing economies of these countries are experiencing rapid urbanization, globalization, increasing demand for and use of fuels, and other factors that are likely to propagate air pollution in all forms. Therefore, it is necessary to assess the level, source, and effects of air pollution in these countries and reflect the findings to guide global and country-specific policies to prevent air pollution and mitigate its effects.

Against this backdrop, this study aims to discuss the health effects of air pollution across genders, age groups, and diseases (i.e. communicable and non-communicable) in the BRICS countries in a comparative global perspective based on the global burden of disease study. The paper’s structure is as follows: Sect. 2 presents data and methods, Sects. 3 & 4 presents the results and discussion, followed by Sect. 5, which presents the conclusions and policy suggestions.

## Methodology

This study utilized data from the global burden of disease (GBD) study report published by the Institute of Health Metrics Evaluation at the University of Washington (IHME 2020). The GBD study is a comprehensive dataset that provides information on the impact of diseases, injuries, and risk factors across age groups, genders, countries, regions, and time. Decision-makers, health sector leaders, researchers, and informed citizens can compare their countries' health progress with that of others and identify the leading causes of health loss that could potentially be avoided by mitigating environmental risk factors. The GBD is regularly updated and provides estimates for all-cause mortality, deaths by cause, years of life lost due to premature mortality (YLLs), years lived with disability (YLDs), and disability-adjusted life years (DALYs). The GBD collaborative network collects data from hospitals, governments, surveys, and other databases worldwide. The data is then cleaned, sorted, and modeled to generate estimates for locations and years.

The methodologies employed in constructing the global burden of disease (GBD) dataset by the Institute for Health Metrics and Evaluation (IHME) are indeed sophisticated, drawing on a blend of empirical data collection, statistical modeling, and computational techniques (Murray et al. 2012; Behera et al. 2022). IHME leverages a diverse array of data sources including vital registration systems, censuses, surveys, hospital records, and scientific literature to gather data on mortality, morbidity, risk factors, and other health indicators (Gakidou et al., 2017).

Sophisticated statistical models such as the Bayesian meta-regression tool DisMod-MR are integral to estimating disease prevalence, incidence, and mortality rates, as they integrate data from multiple sources while accounting for uncertainty and bias (Salomon et al., 2012). Additionally, IHME applies adjustments and standardization techniques to ensure comparability across different data sources and populations, encompassing age standardization and corrections for underreporting and misclassification (Murray et al. 2013).

The GBD database offers numerous merits. Firstly, it provides comprehensive and standardized estimates of health metrics across countries, regions, and time periods, facilitating global comparisons and trend analysis vital for policymakers, researchers, and public health practitioners (Murray et al. 2015). Secondly, IHME's disaggregation of data by age, sex, location, and cause provides detailed insights into health disparities and inequities, enabling targeted interventions and resource allocation (Gakidou et al., 2017). Lastly, IHME’s transparency about methodologies, data sources, and assumptions enhances the credibility and reliability of GBD estimates within the scientific community, facilitating reproducibility and peer review (GBD 2019 Collaborators 2020).

However, the GBD dataset also faces shortcomings. It heavily relies on data availability, quality, and completeness, which can vary significantly across countries and regions, leading to potential uncertainty and bias in estimates, especially in low- and middle-income countries (Foreman et al. 2012). Moreover, GBD estimates may pose interpretation challenges, particularly when comparing different diseases, risk factors, or regions, due to differences in disease definitions, diagnostic criteria, and health systems (Murray et al. 2015). Lastly, health data's dynamic nature necessitates periodic updates and revisions to capture real-time or emerging health trends, as demographic shifts, epidemiological transitions, and changes in healthcare practices evolve over time (Roth et al. 2017).

In conclusion, while the GBD dataset offers valuable insights into global health metrics, users must be aware of its limitations and uncertainties, necessitating critical interpretation and contextual understanding in decision-making and policy development.

**Fig. 1:**
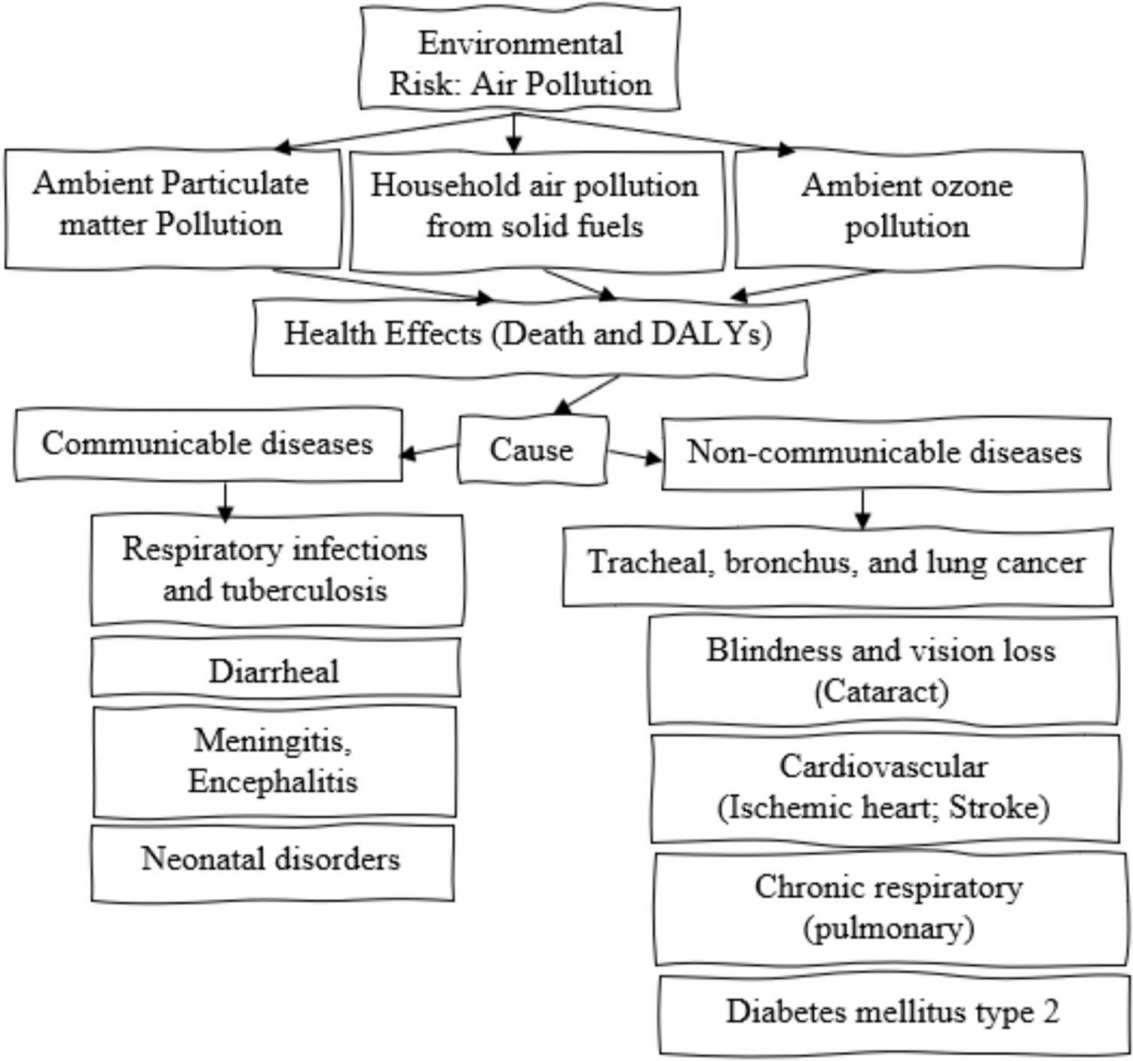
Risk of Air Pollution on Global Health. Source: Author’s conceptualization from the GBD-2019 Survey (IHME 2020)

This study utilized the latest round of the GBD survey-2019, which provides information on 369 diseases and 87 risk factors that can cause death and DALYs. The main focus of this study is to examine the health effects of air pollution. We obtained GBD data from the global health data exchange (GHDx) online database, using GBD tools of IHME. In Fig. 1, we have presented the structure of this study, which illustrates the relationship between health and air pollution. The flow diagram was designed by exploring the GBD dataset. Our findings show that air pollution is one of the environmental risk factors, with the three main sources being ambient particulate matter pollution, household air pollution from solid fuels, and ambient ozone pollution.

Air pollution has severe health effects, which we measure as the disease burden, including the number of deaths, death rate (per 100,000 population), DALYs number, and DALYs rate (per 100,000 population). We have discovered that there are two causes of death and DALYs, which are communicable and non-communicable diseases (NCDs). Ambient particulate matter pollution and household pollution from solid fuels cause various communicable diseases such as respiratory infections, tuberculosis, meningitis, encephalitis, and neonatal disorders. Air pollution also causes several NCDs, including tracheal, bronchus, and lung cancer, cardiovascular disease (i.e. ischemic heart and stroke), diabetes mellitus type 2, chronic respiratory (chronic obstructive pulmonary), and blindness and vision loss (i.e. cataract).

We conducted data analysis using the standard formula for calculating death incidence and DALYs from 1990 to 2019. Age-specific, cause-specific, and gender-wise death and DALY rates were calculated using appropriate numerator and denominator. Four indicators of disease measurement were used, including death rate (deaths per 100,000 population), death numbers (number of deaths in the population), DALYs numbers (number of DALYs in the population), and DALYs rate (DALYs per 100,000 population). After collecting and screening the data, we identified air pollution as a risk factor and the cause of diseases in the sample countries (refer to Fig. 1). This study discusses the cause-specific mortality and DALYs in Brazil, Russia, China, India, and South Africa (BRICS) and compares it with the global scenario to identify the effects of air pollution-related risk factors and their impact on various disease outcomes.

## Results

### ***All-cause***^1^*** death and DALY attributable to air pollution***

**Fig. 2:**
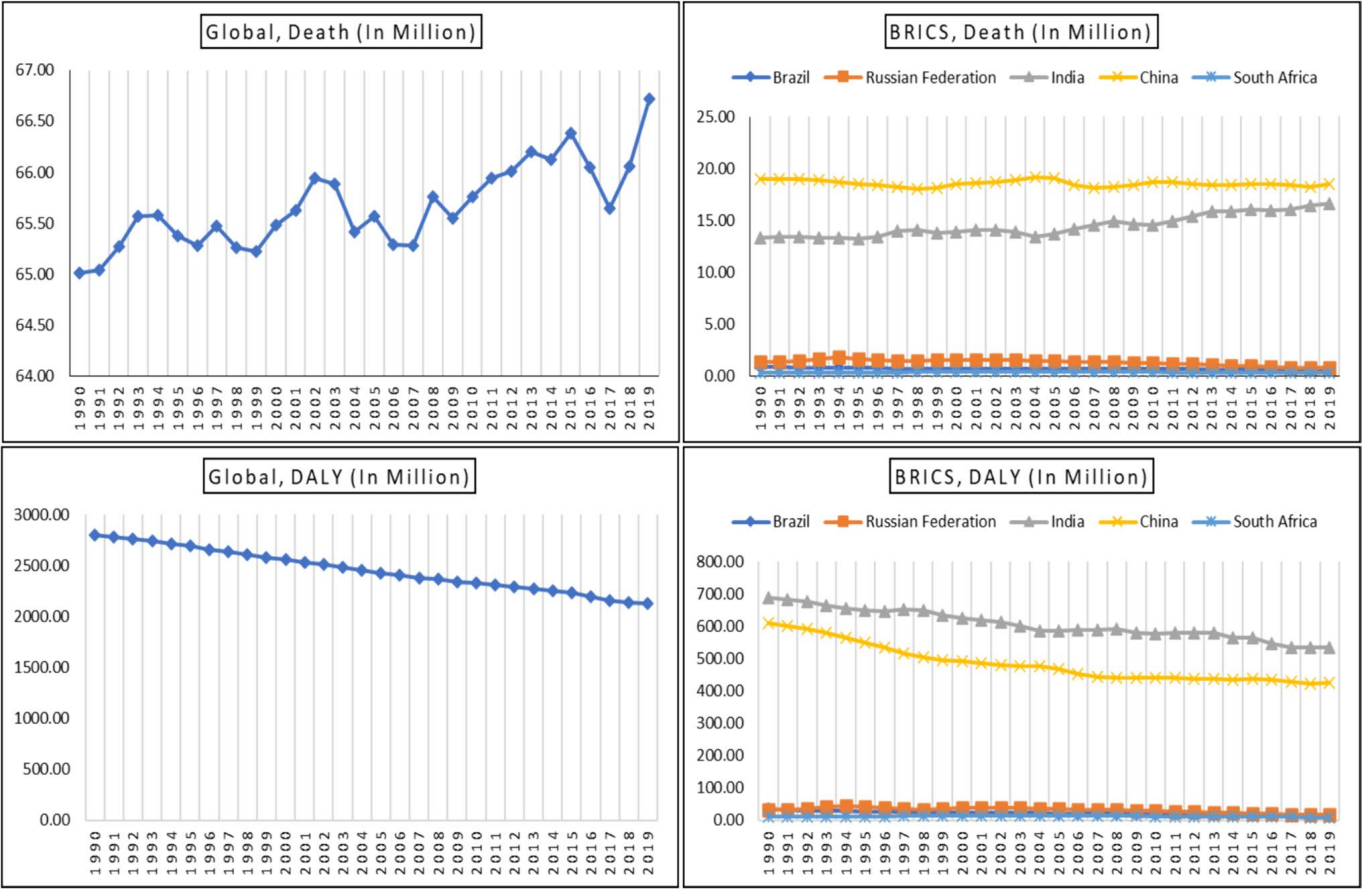
All causes of Death and DALYs number in millions (all ages) attributable to air pollution in Global and BRICS (1990–2019). Note: All causes of Death and DALYs rate (all ages) attributable to air pollution in Global and BRICS are reported in the Appendix Fig. 3. Source: Author’s estimation from the GBD-2019 Survey data (IHME 2020)

Figure 2 illustrates the impact of air pollution on health, in terms of deaths and DALYs, in BRICS from 1990 to 2019. The global death toll due to air pollution was 66.72 million in 2019, which was a 3% increase from 65.01 million in 1990. In India, the number of deaths due to air pollution was 15.41 million in 2019, which was a considerable increase of over 15% from 13.33 million in 1990. On the other hand, deaths caused by air pollution in China declined slightly by 2.63% from 18.96 million in 1990 to 18.48 million in 2019. Brazil, the Russian Federation, and South Africa had much lower death rates due to air pollution compared to China and India. In addition, Fig. 2 compares the DALY trends globally with the BRICS countries. It shows that the number of DALYs worldwide declined from 2807.18 million in 1990 to 2132.85 million in 2019. The same trend was also observed in the BRICS countries.

In the appendix, Fig. 5 shows the trends of global death rates and DALY rates caused by air pollution over the years. The data reveals that the death rate declined from 122 to 86 per 100,000 persons worldwide between 1990 and 2019. Similarly, China and India also witnessed a decline in death rates due to air pollution from 160 and 156 in 1990 to 130 and 120 in 2019, respectively. This trend is also observed in other BRICS countries. Additionally, Figure 5 presents the DALY rates of both global and BRICS countries, which also saw a decline from 5247 persons in 1990 to 2757 in 2019. The declining trend of air pollution risk towards human health can be attributed to several factors at the global and BRICS levels, including CO2 emissions per capita, PM2.5 air pollution exposure, renewable energy consumption, and fossil fuel energy consumption (See Figure 6 and 7. Despite Brazil having higher levels of CO2 emissions per capita and PM2.5 air pollution exposure, their death and DALY rates remain much lower compared to India and China. However, it is essential to adopt clean energy and healthcare facilities to minimize the adverse effects of air pollution on human health.

### Communicable and non-communicable diseases attributable to air pollution

The presented data in Table 1 highlights the impact of air pollution on communicable and non-communicable diseases (CDs and NCDs) globally and in BRICS countries. Air pollution was divided into three minor components, namely ambient particulate matter pollution, household air pollution from solid fuels, and ambient ozone pollution. In 2019, the global death toll due to CDs and NCDs caused by air pollution was 11.36 million and 55.36 million respectively. In India, the deaths due to air pollution in case of CDs and NCDs were 2.76 million and 13.91 million persons respectively in 2019. Similarly, in China, the figures were 0.53 million and 17.95 million persons respectively during the same period. The data in Table 1 also shows that the BRICS countries have experienced a transition from CDs to NCDs, with CDs dominating in 1990, especially in China and India. However, their prevalence has not decreased over the years. The major contributor to NCDs is ambient particulate matter pollution, which resulted in 8.25 million and 13.82 million deaths in various NCDs in 2019 in India and China respectively. We also found that ambient ozone pollution does not cause any communicable diseases, rather it affects NCDs related deaths, which was around 3.65 million at the global level in 2019.

**Table 1:** Death and DALYs number in millions (all ages) attributable to air pollution of communicable and non-communicable diseases in Global and BRICS (1990–2019)

Risk	Location	Communicable disease				Non-communicable disease			
		Death number (In Million)		DALY number (in million)		Death number (in million)		DALY number (in million)	
		1990	2019	1990	2019	1990	2019	1990	2019
Air pollution	Global	21.66	11.36	1688.89	720.50	43.36	55.36	1118.29	1412.34
	Brazil	0.24	0.08	17.27	3.12	0.63	0.52	17.83	13.92
	Russia	0.03	0.02	2.12	0.73	1.32	0.76	31.05	16.93
	India	5.91	2.76	474.26	162.67	7.41	13.91	214.33	372.34
	China	3.05	0.53	225.55	15.95	15.91	17.95	383.51	409.14
	South Africa	0.12	0.07	8.95	3.97	0.13	0.22	4.14	6.17
Ambient particulate matter pollution	Global	4.05	4.66	296.86	258.22	16.42	36.75	407.93	923.93
	Brazil	0.06	0.06	3.87	2.15	0.24	0.37	6.94	10.08
	Russia	0.03	0.41	1.82	0.71	1.16	0.72	27.33	16.17
	India	1.12	1.55	89.41	89.10	1.67	8.25	47.94	222.31
	China	0.72	0.02	51.87	11.86	4.48	13.82	108.40	316.77
	South Africa	0.05	0.06	3.52	3.26	0.07	0.19	2.22	5.18
Household pollution from solid fuels	Global	17.60	6.70	1392.03	462.28	25.98	16.44	692.44	452.47
	Brazil	0.18	0.02	13.40	0.98	0.38	0.12	10.76	3.27
	Russia	0.00	0.12	0.30	0.03	0.13	0.02	3.10	0.54
	India	4.79	1.21	384.85	73.58	5.62	4.86	163.78	135.41
	China	2.33	0.00	173.67	4.09	10.97	3.51	266.66	83.32
	South Africa	0.07	0.01	5.43	0.72	0.06	0.03	1.90	0.90
Ambient ozone pollution	Global	No risk	No risk	No risk	No risk	2.07	3.65	39.71	13.73
	Brazil	No risk	No risk	No risk	No risk	0.01	0.04	0.17	0.63
	Russia	No risk	No risk	No risk	No risk	0.04	0.01	0.72	0.23
	India	No risk	No risk	No risk	No risk	0.43	1.68	9.21	30.63
	China	No risk	No risk	No risk	No risk	1.07	0.93	19.73	62.10
	South Africa	No risk	No risk	No risk	No risk	0.002	0.01	0.03	0.12

Note: These are estimated values. We have not reported the uncertainty interval i.e. lower and upper limits of the estimated values due to limited space in the table. No risk = Communicable diseases have no risk for ambient ozone pollution as per the GBD data and it affects only the cause of non-communicable diseases which includes Chronic obstructive pulmonary disease. We have used 1990 as a reference period for our analysis. Deaths and DALYs number attributable to air pollution due to communicable diseases is the sum of two components ambient particulate matter pollution, and household air pollution from solid fuels; Deaths and DALYs number attributable to air pollution due to non-communicable diseases are the sum of three components ambient particulate matter pollution, household air pollution from solid fuels, and Ozone pollution. Death and DALYs rate (all ages) attributable to air pollution of communicable and non-communicable diseases in Global and BRICS is reported in Appendix Table 4. Source: Author’s estimation from the GBD-2019 Survey data (IHME 2020)

In Table 1, you can find the DALY numbers caused by air pollution for CDs and NCDs. The data shows that the DALYs have increased for NCDs while they have decreased for CDs globally, as well as in BRICS. In 1990, the global DALY number for CDs was 1688.89 million, and by 2019, it decreased to 720.50 million. Whereas, in 1990, the global DALY number for NCDs was 1118.29 million, and by 2019, it decreased to 1412.34 million. Similar results were noticed in the case of air pollution's minor risk factors in BRICS countries. You can also find death and DALYs rates of people affected by CDs and NCDs due to air pollution in Appendix Table 4. The table presents data for global and BRICS country contexts between 1990 and 2019. According to the table, in 2019, the CDs death rate was 15 (CI:12–18), and the DALYs rate was 931 (CI: 761–1130) at the global level. Whereas, the NCDs death rate was 72 (CI: 63–80), and the DALYs rate was 1825 (CI: 1611–2030) in 2019. The trend of increasing NCDs as compared to CDs has been seen in most of the BRICS countries.

Air pollution has evolved in its impact on health, initially affecting communicable diseases (CDs) more prominently but now increasingly affecting Non-communicable diseases (NCDs) (Guan et al. 2016). Recent research highlights this shift, emphasizing the growing influence of air pollution on NCDs compared to previous decades. In BRICS countries, air pollution significantly contributes to NCD-related deaths, with ambient particulate matter pollution and household air pollution from solid fuels being major contributors. Globally, ambient particulate matter pollution caused approximately 37 million deaths in 2019, while household air pollution resulted in about 16.44 million NCD-related deaths during the same period. Notably, India and China bear the highest burden of NCD-related deaths attributed to ambient ozone pollution, indicating a pressing need for mitigation efforts in these regions (See Table 1 and Appendix Table 4). These findings underscore the substantial public health burden posed by air pollution, particularly in China and India, underscoring the urgency for effective mitigation strategies.

A significant observation is the decline in the contribution of household air pollution from solid fuels to non-communicable disease (NCD)-related deaths and disability-adjusted life years (DALYs) globally and within BRICS countries between 1990 and 2019. Notably, in China, the number of NCD-related deaths attributed to household solid fuels decreased from 10.97 million in 1990 to 3.51 million in 2019 (See Table 1 and Appendix Table 4). Conversely, the prevalence of NCD-related deaths and DALYs due to ambient particulate matter pollution remains a critical issue across almost all BRICS countries, posing a substantial risk to global health.

### Gender-wise health effects of air pollution

**Fig. 3:**
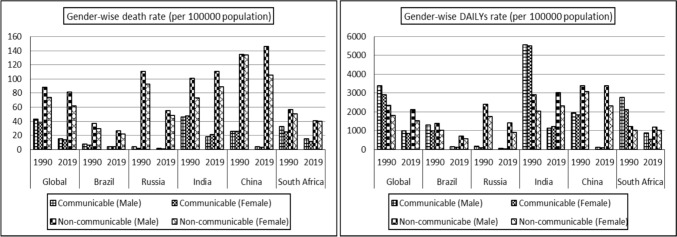
Gender-wise Death and DALY rates (per 100,000 population) for all ages attributable to air pollution. Source: Author’s estimation from the GBD-2019 Survey data (IHME 2020)

In Fig 3, we can see the impact of air pollution on the health of males and females in the BRICS countries and globally between 1990 and 2019. The health effects are measured by the number of deaths and DALYs (disability-adjusted life years) per 100,000 population, for communicable diseases (CDs) and non-communicable diseases (NCDs). The figure shows that males have a higher death rate in both CDs and NCDs compared to females, both in the BRICS countries and globally. The same trend is observed in DALYs rates. Among the BRICS countries, China has the highest number of NCD-related deaths and DALYs, followed by India.

The data presented in Fig 3 suggests that males bear a greater burden of ambient particulate matter pollution, likely due to their increased exposure to outdoor environments. In contrast, females, who predominantly spend more time indoors, appear to be less vulnerable. This discrepancy may be attributed to the reduction in household air pollution from solid fuels over time, potentially rendering females less susceptible to air pollution compared to males (Murray et al. 2020).

### Age-wise health effects of air pollution risk factors

**Fig. 4:**
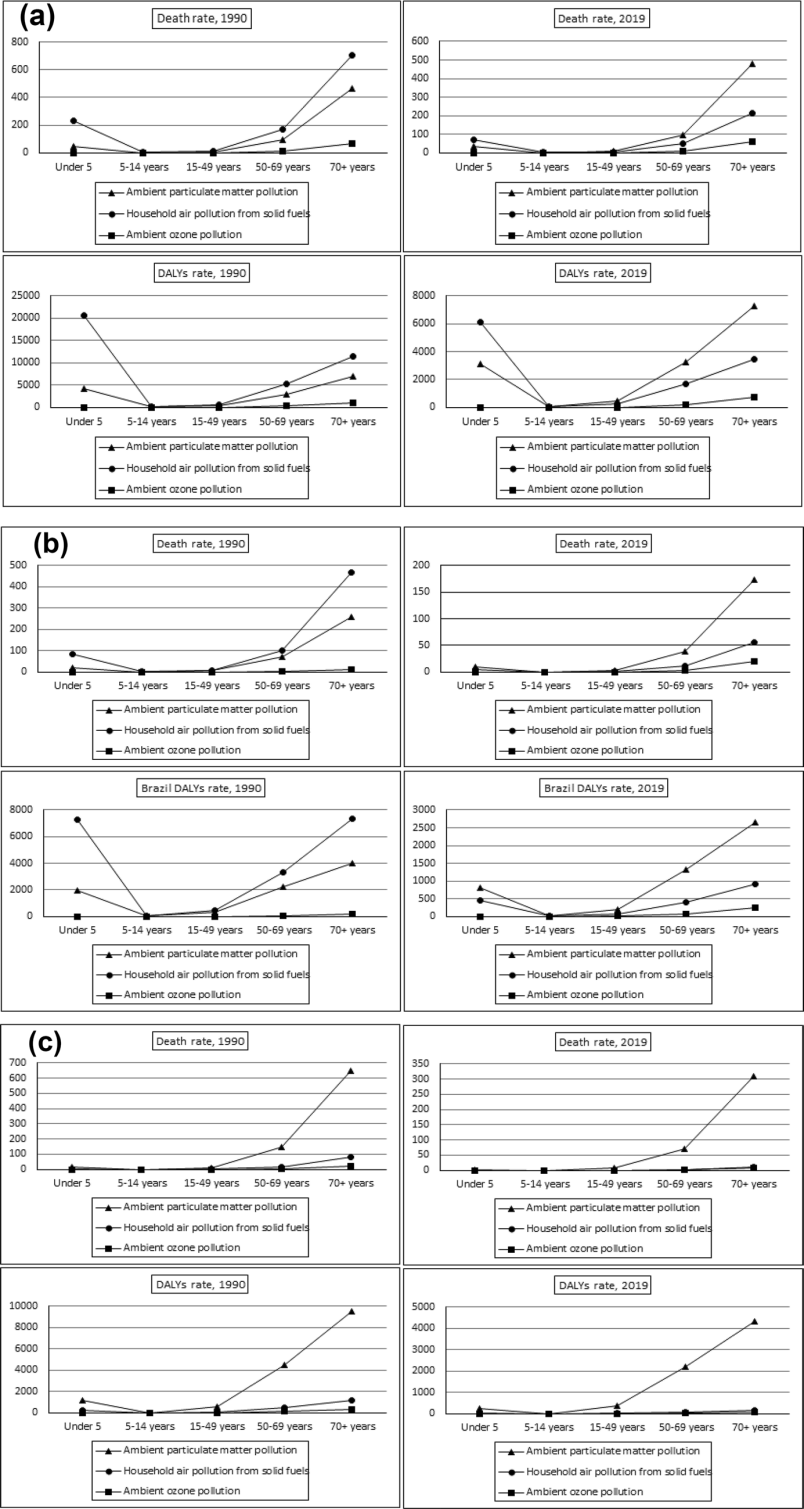

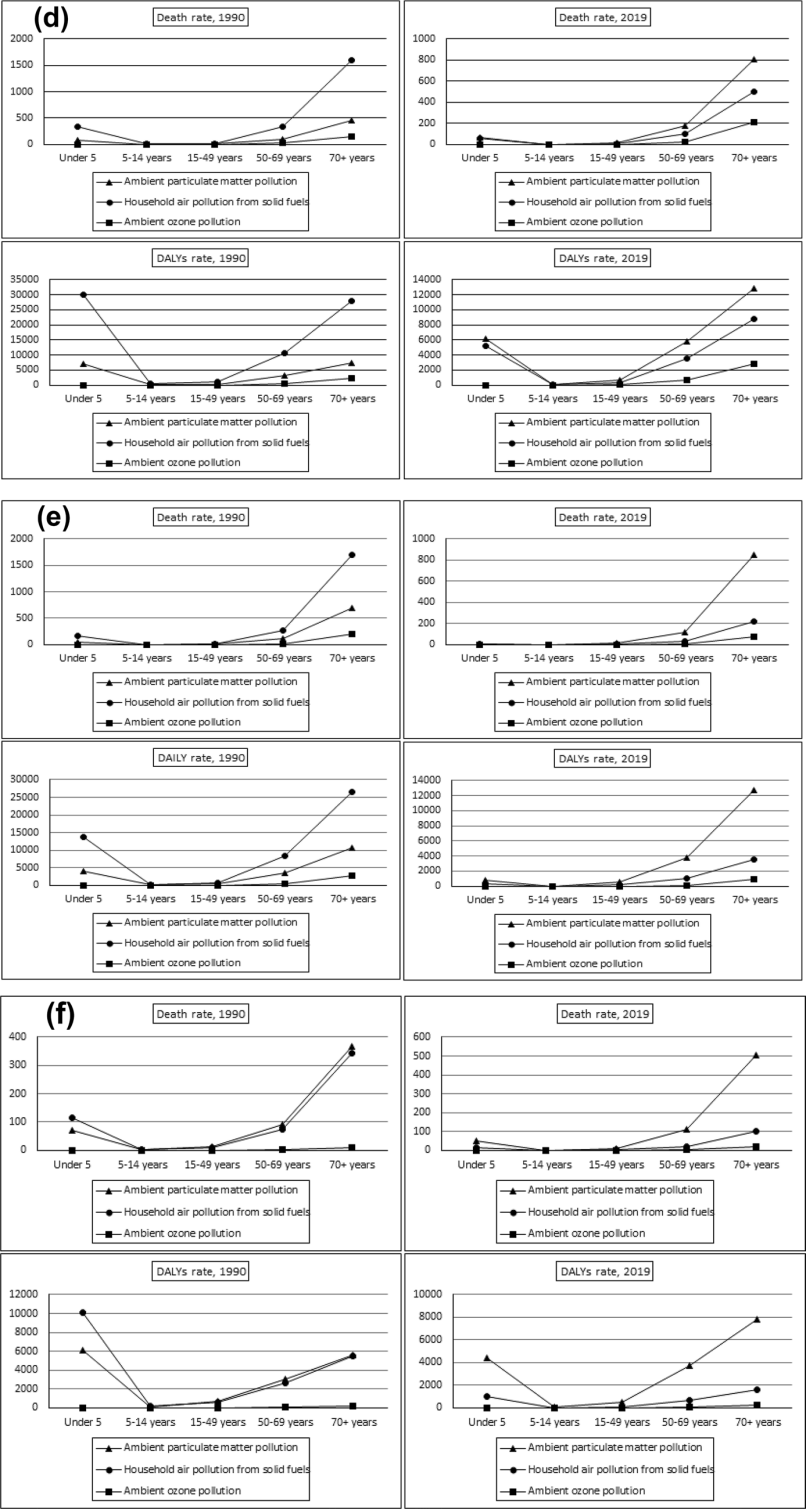
**a** Age-wise total death and DALYs rate attributable air pollution risk factors in Global. **b** Age-wise total death and DALYs rate attributable air pollution risk factors in Brazil. **c** Age-wise total death and DALYs rate attributable air pollution risk factors in Russia. **d** Age-wise total death and DALYs rate attributable air pollution risk factors in India. **e** Age-wise total death and DALYs rate attributable air pollution risk factors in China. **f** Age-wise total death and DALYs rate attributable air pollution risk factors in South Africa. Source: Author’s estimation from the GBD-2019 Survey data (IHME 2020)

**Fig.5:**
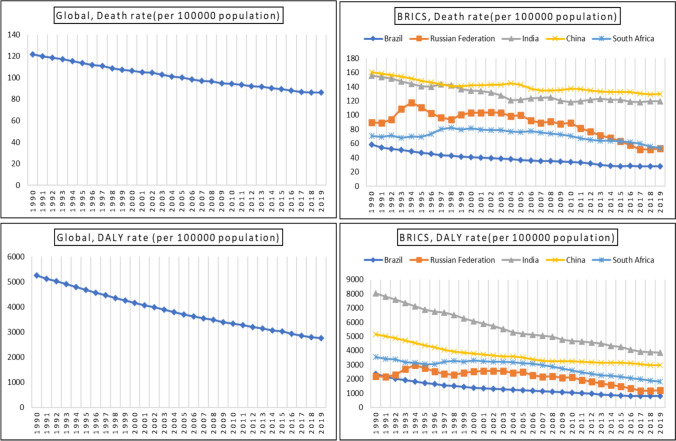
All causes of Death and DALYs rate (per 100,000 population) (all ages) attributable to air pollution in Global and BRICS (1990–2019). Source: Author’s estimation from the GBD-2019 Survey data (IHME 2020)

**Fig.6:**
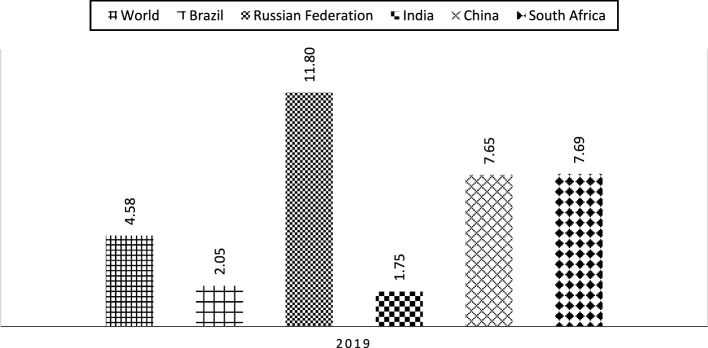
CO2 emissions (metric tons per capita) in Global and BRICS. Source: Author’s estimation from World Developpment Indicators data, World Bank

**Fig.7:**
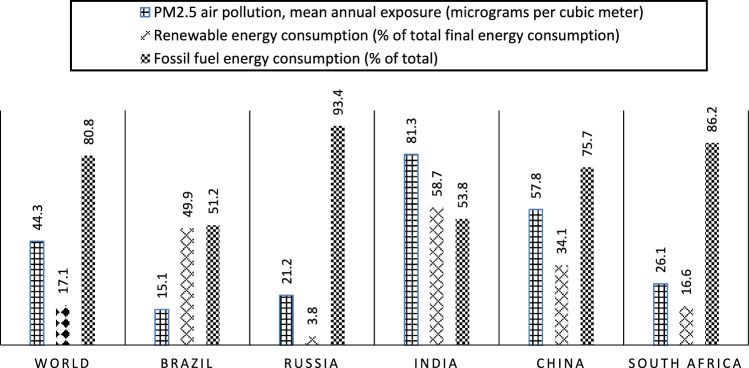
A3 Air Pollution Exposure in Global and BRICS, 2019. Source: Author’s estimation from World Developpment Indicators data, World Bank

The impact of air pollution on human health across different age groups is shown in Fig. 4a–f. The study divides age groups into sub-groups such as under-5 years, 5–14 years, 15–49 years, 50–69 years, and 70+ years. Fig. 4 a shows the death and DALYs rates for different age groups globally. It indicates that indoor air pollution was the primary risk factor for deaths and DALYs in the 1990s, followed by ambient particulate matter and ambient ozone pollution.

The impact of air pollution on different age groups in the 1990s showed that it was more severe in the 50–69 years and 70+ years age groups, followed by children under 5 years. However, the effects were less severe among the age group 5–14 years and 15–69 years. Our study shows that the impact of air pollution on human health follows a 'U' shaped curve, initially affecting the under-5 year age group, and then becoming more severe among older age populations. This finding was consistent across China, Russia, India, and South Africa. The effects of ambient ozone pollution were negligible in the 1990s, but its impact on population deaths and DALYs had increased in the 70+ year population in China, Russia, and India. As compared to the 1990 GBD study, the effects of air pollution were mostly impacting the population above 50 years in the 2019 GBD estimations. Our study, like that of Saldiva et al. (1995a, b), found a strong link between higher levels of particulate matter in the air and increased mortality rates among the elderly. This highlights the urgent need to tackle air pollution to safeguard vulnerable groups such as the elderly from adverse health effects.

### Disease-wise health effects of air pollution on global deaths

Table 2 displays the number of deaths that can be attributed to air pollution in the cases of various communicable diseases (CDs) and non-communicable diseases (NCDs) in both the global and BRICS country contexts. Our findings indicate that air pollution, in the form of ambient particulate matter pollution and household pollution from solid fuels, contributed to the occurrence of five communicable diseases, namely diarrhea, meningitis, encephalitis, neonatal disorders, respiratory infections, and tuberculosis. In addition, six non-communicable diseases were found to be linked to air pollution, including Tracheal, bronchus, and lung cancer, ischemic heart disease, stroke, chronic obstructive pulmonary disease, diabetes mellitus type 2, and chronic obstructive pulmonary disease.

**Table 2:** Death number in millions (all ages) attributable to air pollution on various communicable and non-communicable diseases in Global and BRICS (1990–2019)

Risk	Cause	Global		Brazil		Russia		India		China		South Africa	
		1990	2019	1990	2019	1990	2019	1990	2019	1990	2019	1990	2019
	Communicable diseases												
Air pollution	Diarrheal	0.48	0.10	0.002	0.00003	0.0001	0.00001	0.16	0.02	0.01	0.0002	0.002	0.0002
	Meningitis	0.07	0.03	0.0004	0.00002	0.0001	0.00001	0.01	0.004	0.003	0.0002	0.0002	0.0001
	Encephalitis	0.005	0.003	0.00003	0	0.00002	0.00001	0.003	0.001	0.0004	0.0001	0	0
	Neonatal disorders	6.04	3.73	0.08	0.02	0.01	0.002	1.95	0.87	0.44	0.06	0.04	0.02
	Respiratory infections and tuberculosis	15.05	7.49	0.15	0.07	0.02	0.018	3.79	1.86	2.60	0.47	0.08	0.05
Ambient particulate matter pollution	Diarrheal	0.07	0.03	0.0005	0.00002	0.0001	0.00001	0.03	0.01	0.002	0.0001	0.001	0.0002
	Meningitis	0.01	0.01	0.0001	0.00001	0.0001	0.00001	0.002	0.002	0.001	0.0001	0.0001	0.0001
	Encephalitis	0.001	0.001	0.00001	0	0.00002	0.00001	0.0005	0.001	0.0001	0.0001	0	0
	Neonatal disorders	1.07	1.35	0.02	0.01	0.01	0.002	0.35	0.48	0.10	0.04	0.02	0.02
	Respiratory infections and tuberculosis	2.90	3.26	0.04	0.05	0.02	0.02	0.74	1.06	0.62	0.37	0.03	0.04
Household air pollution from solid fuels	Diarrheal	0.41	0.07	0.002	0.00001	0.0000	0	0.13	0.01	0.01	0.0001	0.001	0.00005
	Meningitis	0.06	0.02	0.0003	0.00001	0.00002	0	0.01	0.002	0.002	0.0001	0.0001	0.00001
	Encephalitis	0.004	0.001	0.00002	0	0	0	0.002	0.001	0.000	0.00002	0	0
	Neonatal disorders	4.98	2.37	0.06	0.01	0.002	0.00009	1.60	0.40	0.34	0.02	0.03	0.004
	Respiratory infections and tuberculosis	12.15	4.23	0.11	0.02	0.003	0.00056	3.05	0.81	1.99	0.11	0.05	0.01
	Non-communicable diseases												
Air pollution	Tracheal, bronchus, and lung cancer	2.20	3.87	0.03	0.04	0.08	0.04	0.10	0.28	0.84	2.09	0.01	0.02
	Ischemic heart	12.25	18.43	0.22	0.17	0.65	0.41	2.36	4.87	2.00	4.64	0.04	0.06
	Stroke	13.79	17.02	0.24	0.14	0.49	0.25	1.66	2.71	5.44	6.79	0.05	0.07
	Chronic obstructive pulmonary	14.92	14.59	0.10	0.10	0.10	0.04	3.41	6.29	8.07	4.36	0.02	0.03
	Diabetes mellitus type 2	1.31	2.93	0.04	0.07	0.01	0.02	0.19	0.63	0.16	0.40	0.02	0.05
Ambient particulate matter pollution	Tracheal, bronchus, and lung cancer	1.18	3.08	0.01	0.03	0.07	0.04	0.03	0.19	0.28	1.71	0.01	0.01
	Ischemic heart	6.08	13.32	0.09	0.13	0.58	0.39	0.61	3.29	0.61	3.74	0.02	0.05
	Stroke	5.08	11.43	0.09	0.11	0.44	0.24	0.37	1.64	1.62	5.42	0.02	0.05
	Chronic obstructive pulmonary	3.52	6.95	0.03	0.05	0.06	0.03	0.62	2.71	1.92	2.64	0.01	0.02
	Diabetes mellitus type 2	0.56	1.97	0.02	0.06	0.01	0.02	0.04	0.42	0.05	0.32	0.01	0.05
Household air pollution from solid fuels	Tracheal, bronchus, and lung cancer	1.02	0.80	0.02	0.01	0.01	0.001	0.08	0.09	0.57	0.37	0.004	0.002
	Ischemic heart	6.18	5.11	0.13	0.04	0.07	0.01	1.75	1.58	1.38	0.90	0.02	0.01
	Stroke	8.71	5.59	0.15	0.04	0.05	0.01	1.29	1.07	3.83	1.37	0.02	0.01
	Chronic obstructive pulmonary	9.32	3.98	0.06	0.02	0.01	0.001	2.35	1.90	5.08	0.79	0.01	0.003
	Diabetes mellitus type 2	0.75	0.96	0.03	0.02	0.001	0.001	0.15	0.21	0.11	0.08	0.01	0.01
Ozone	Chronic obstructive pulmonary	2.07	3.65	0.01	0.04	0.04	0.01	0.43	1.68	1.07	0.93	0.002	0.01

Note: We have not reported the uncertainty interval i.e. lower and upper limits of the estimated values due to limited space in the table. We have used 1990 as a reference period for our analysis. Deaths number attributable to air pollution due to communicable diseases are the sum of two components ambient particulate matter pollution, and household air pollution from solid fuels; Deaths number attributable to air pollution due to non-communicable diseases are the sum of three components ambient particulate matter pollution, household air pollution from solid fuels, and Ozone pollution. Source: Author’s estimation from the GBD-2019 Survey data (IHME 2020)

The data reveals that globally, respiratory infections and tuberculosis were responsible for the highest number of deaths (15.05 million) followed by chronic obstructive pulmonary disease (14.92 million), stroke (13.79 million), and ischemic heart disease (12.25 million) in the 1990s. However, there has been a shift towards a rising number of deaths due to non-communicable diseases, such as ischemic heart disease (18.43 million), stroke (17.02 million), and chronic obstructive pulmonary disease (14.59 million) in 2019. Hence, our analysis suggests that there has been an epidemiological transition from communicable diseases (CDs) to non-communicable diseases (NCDs) at a global level. This conclusion has been supported by several country-level studies (Zhou et al. 2019; Quigley 2006; Adogu et al., 2015).

Air pollution has a significant impact on public health in the BRICS countries, with different diseases affected in different ways. In China, for instance, stroke (6.79 million) and chronic obstructive pulmonary disease (4.36 million) were the leading causes of death in 2019. Non-communicable diseases (NCDs) such as tracheal, bronchus, and lung cancer; ischemic heart disease; stroke; and type 2 diabetes have increased since the 1990s. Meanwhile, in Brazil, cardiovascular diseases such as ischemic heart disease (0.22 million) and stroke (0.24 million) were the top causes of death in the 1990s, but these have declined in 2019. However, in India, the burden of disease due to air pollution has increased in 2019 compared to the 1990s. Respiratory infection and tuberculosis (3.79 million) were the leading causes of death in the 1990s, followed by pulmonary disease (3.41 million), but in 2019, pulmonary disease (6.29 million) was the top contributor, followed by ischemic heart disease (4.87 million) and stroke-related cardiovascular diseases (2.71 million). Similar trends were observed in South Africa, where cardiovascular diseases have declined, but new types of NCDs have emerged, including type 2 diabetes (0.05 million), pulmonary disease (0.03 million), and tracheal, bronchus, and lung cancer (0.02 million), due to air pollution.

It has been discovered that two contagious diseases caused by air pollution were widespread in the global disease burden during the 1990s. These diseases are neonatal disorders which accounted for 6.04 million cases, and respiratory infections and tuberculosis, which accounted for 15.05 million cases. The major contributor to these diseases in 1990 was household air pollution from solid fuels, but this contribution has decreased since then. In 2019, air pollution was responsible for 7.49 million deaths due to respiratory infections and tuberculosis, and 3.73 million deaths due to neonatal disorders, globally. In India alone, air pollution caused around 1.86 million deaths due to respiratory infections and tuberculosis in 2019. There has been a substantial reduction in deaths caused by many communicable diseases, particularly those originating from household air pollution, since 1990. However, ambient particulate matter pollution remains a significant risk factor for many non-communicable diseases globally, including in BRICS. Several NCDs have emerged as a significant source of the global burden of disease, such as Ischemic heart, Tracheal, bronchus, and lung cancer, Stroke, Chronic obstructive pulmonary, and Diabetes mellitus type 2.

### Disease-wise health effects of air pollution on Global DALYs

Table 3 provides a comparative analysis of the disease-wise total numbers of disability-adjusted life years (DALYs) attributable to air pollution for all age groups in the world and the BRICS countries. It is observed that the overall number of DALYs due to communicable diseases (CDs) such as respiratory infections and tuberculosis have decreased globally in 2019 as compared to the 1990s. Neonatal disorders also showed a decline (331 million). However, the numbers for non-communicable diseases (NCDs) such as type 2 diabetes mellitus (129 million), ischemic heart disease (453 million), and stroke (434 million) have increased.

**Table 3:** DALYs numbers in millions (all ages) attributable to air pollution of various communicable and non-communicable diseases in Global and BRICS (1990–2019)

Risk	Cause	Global		Brazil		Russia		India		China		South Africa	
		1990	2019	1990	2019	1990	2019	1990	2019	1990	2019	1990	2019
	Communicable diseases												
Air pollution	Diarrheal	43.17	9.29	0.20	0.003	0.01	0.001	14.31	1.88	0.68	0.02	0.17	0.02
	Meningitis	6.56	2.99	0.03	0.001	0.01	0.001	0.93	0.33	0.23	0.02	0.02	0.01
	Encephalitis	0.43	0.25	0.002	0.000	0.002	0.001	0.23	0.11	0.04	0.01	0.0004	0.0002
	Neonatal disorders	537.29	331.26	7.05	1.59	0.96	0.17	173.51	77.59	38.93	5.36	3.77	2.11
	Respiratory infections and tuberculosis	1101.43	376.71	9.98	1.53	1.13	0.56	285.29	82.75	185.67	10.55	4.99	1.84
Ambient particulate matter	Diarrheal	6.52	2.84	0.04	0.002	0.01	0.001	2.50	0.99	0.14	0.01	0.06	0.02
	Meningitis	0.87	0.78	0.01	0.001	0.01	0.001	0.18	0.17	0.05	0.01	0.01	0.01
	Encephalitis	0.09	0.12	0.001	0.000	0.002	0.0005	0.04	0.06	0.01	0.01	0.0002	0.0002
	Neonatal disorders	94.70	120.28	1.54	1.01	0.81	0.16	31.54	42.35	8.97	3.87	1.44	1.71
	Respiratory infections and tuberculosis	194.68	134.20	2.28	1.13	0.99	0.55	55.14	45.52	42.70	7.96	2.01	1.52
Solid fuels from household	Diarrheal	36.66	6.46	0.16	0.001	0.002	0.00004	11.81	0.89	0.54	0.01	0.11	0.004
	Meningitis	5.70	2.21	0.02	0.0005	0.002	0.00004	0.74	0.16	0.18	0.005	0.01	0.001
	Encephalitis	0.33	0.13	0.002	0.0002	0.0003	0.00002	0.18	0.05	0.03	0.002	0.0003	0.00004
	Neonatal disorders	442.59	210.98	5.51	0.57	0.15	0.01	141.97	35.24	29.96	1.49	2.33	0.39
	Respiratory infections and tuberculosis	906.75	242.51	7.70	0.40	0.14	0.02	230.14	37.24	142.97	2.59	2.97	0.32
	Non-communicable diseases												
Air pollution	Tracheal, bronchus, and lung cancer	57.55	89.52	0.73	0.84	2.25	1.06	2.85	7.14	22.80	47.07	0.30	0.41
	Ischemic heart	314.78	453.54	5.93	4.40	14.71	8.56	70.86	133.32	54.01	100.34	1.06	1.48
	Stroke	363.13	434.75	7.05	3.72	11.04	5.50	46.94	73.09	140.98	160.95	1.44	1.74
	Chronic obstructive pulmonary	333.62	309.25	2.25	2.03	2.36	0.88	85.72	137.28	164.85	79.41	0.68	0.86
	Diabetes mellitus type 2	54.57	129.56	1.69	2.83	0.75	0.93	8.84	29.16	9.69	22.50	0.57	1.67
Ambient particulate matter	Tracheal, bronchus, and lung cancer	30.11	70.16	0.31	0.66	2.05	1.03	0.79	4.84	7.38	38.57	0.19	0.36
	Ischemic heart	145.90	321.74	2.52	3.43	13.27	8.30	18.42	90.28	16.60	81.00	0.62	1.29
	Stroke	128.57	287.36	2.71	2.78	9.88	5.32	10.45	44.28	41.81	128.47	0.77	1.47
	Chronic obstructive pulmonary	79.82	154.14	0.69	1.06	1.46	0.63	16.11	63.51	39.70	50.56	0.33	0.64
	Diabetes mellitus type 2	23.30	90.34	0.71	2.16	0.66	0.90	2.12	19.35	2.91	18.16	0.32	1.41
Solid fuels from household	Tracheal, bronchus, and lung cancer	27.44	19.36	0.42	0.18	0.20	0.03	2.06	2.30	15.41	8.49	0.12	0.04
	Ischemic heart	168.88	131.80	3.41	0.97	1.44	0.26	52.43	43.04	37.41	19.34	0.44	0.19
	Stroke	234.56	147.38	4.34	0.95	1.16	0.18	36.49	28.81	99.17	32.48	0.68	0.26
	Chronic obstructive pulmonary	214.09	93.01	1.39	0.34	0.18	0.02	60.40	43.14	105.42	15.12	0.32	0.10
	Cataract	15.46	21.43	0.21	0.16	0.04	0.02	5.48	8.27	2.42	3.54	0.08	0.05
	Diabetes mellitus type 2	31.26	39.22	0.98	0.67	0.08	0.03	6.72	9.81	6.78	4.34	0.25	0.26
ozone	Chronic obstructive pulmonary	39.71	62.10	0.17	0.63	0.72	0.23	9.21	30.63	19.73	13.73	0.03	0.12

Note: We have not reported the uncertainty interval i.e. lower and upper limits of the estimated values due to limited space in the table. We have used 1990 as a reference period for our analysis. DALY number attributable to air pollution due to communicable diseases is the sum of two components ambient particulate matter pollution, and household air pollution from solid fuels; DALY number attributable to air pollution due to non-communicable diseases is the sum of three components ambient particulate matter pollution, household air pollution from solid fuels, and Ozone pollution. Source: Author’s estimation from the GBD-2019 Survey data (IHME 2020)

Similar trends were observed for other countries as well, where DALY numbers increased for various NCDs. For instance, the DALY numbers of Tracheal, bronchus, and lung cancer (7.14 million), stroke (73 million), Chronic obstructive pulmonary disease (137. million), and type 2 diabetes mellitus (29 million) increased in India in 2019 as compared to 1990. China also showed similar trends. However, the DALY numbers of many NCDs had declined in Brazil and Russia in 2019 as compared to 1990, such as stroke and ischemic heart disease, while type 2 diabetes mellitus increased in 2019.

It is found that ambient ozone pollution and ambient particulate matter pollution are the primary contributors to the burden of diseases at the global level as well as in BRICS countries. In 2019, many NCDs were caused by these two risk factors, resulting in deaths and DALYs. In 1990, household air pollution was considered a major risk factor, followed by ambient ozone pollution and ambient particulate matter pollution, leading to more deaths and DALYs due to CDs.

It is observed that the DALY numbers for two major communicable diseases, namely Neonatal disorders, Respiratory infections, and tuberculosis, have increased globally from 1990 to 2019 due to ambient particulate matter pollution. However, the effects of solid fuels from households on these two diseases have declined globally, including India and South Africa.

Almost all NCDs originating from ambient matter pollution have increased globally from 1990 to 2019, including the BRICS countries. According to the GBD data, cataract, a disease leading to blindness and vision loss, has increased in 2019 due to household air pollution from solid fuels.

## Discussion

Over the years, the emergence and growth of the BRICS countries have been influenced by various factors, including environmental, social, economic, and political factors. Despite pre-existing differences, the impact of air pollution on people's health and well-being has remained a major area of academic inquiry and policy interventions (Li et al. 2022; Manisalidis et al. 2020a, b; Kankaria et al. 2014). The effects of air pollution on non-communicable diseases (NCDs) and disorders have been studied extensively, globally, including in the BRICS countries (Badulescu et al. 2019; Balakrishnan et al. 2014; Aguilar-Gomez et al. 2022). This study examines global health trends in deaths and DALYs attributable to air pollution, focusing particularly on the BRICS countries from 1990 to 2019. The study reveals that although communicable diseases such as respiratory infections and tuberculosis contributed to a significant number of deaths and DALYs in the 1990s, the scenario changed since then, with NCDs such as ischemic heart disease and coronary artery disease emerging as the most catastrophic illnesses in recent decades. While global mean exposure to particulate matter air pollution had increased, none of the BRICS countries except India showed a similar trend. Similarly, while the death rate associated with particulate matter air pollution had decreased in Brazil, Russia, India, and South Africa, China was an exception, with a doubling of death rates due to particulate matter air pollution between 1990 and 2019.

Air pollution has been a cause of concern in Brazil, with ambient particulate air pollution leading to high death rates and disability-adjusted life years (DALYs) across all age groups, especially among the elderly population. Studies show that in 1990 and 1991, there was a higher mortality rate among the elderly population due to PM2.5 pollution (Saldiva et al. 1995a, b). Although air pollution has resulted in high mortality rates in Brazil, it still has the lowest mortality and DALY rate among the BRICS countries. Over time, there has been a significant decrease in deaths due to stroke and DALYs related to respiratory infections and tuberculosis. However, studies have shown an increase in respiratory admissions in Brazilian hospitals in the early 2000s due to the rise in ozone or particulate matter air pollution (Braga et al. 2001; Gouveia and Fletcher 2000).

Since 1990, both deaths and DALYs associated with household air pollution and ambient ozone pollution in Russia have been relatively low. However, the number of deaths and DALYs caused by ambient particulate matter air pollution has been high for years. Research shows that particulate matter with an aerodynamic diameter of less than 10 µm is prevalent in the air of urbanized Russian cities with high technogenic load, and this has resulted in adverse health impacts (Barskova et al. 2019). Our study also suggests a higher concentration of deaths among the elderly in Russia and other BRICS countries. It is important to note that the baseline mortality due to various general and contextual factors such as age and higher consumption of alcohol is quite high, which could lead to an overestimation of health impacts caused by air pollution (Orru et al. 2009). Despite a decrease in exposure and population growth in Russia, a net increase in attributable mortality due to air pollution has been persistent (Cohen et al. 2017). Our study shows that the mean annual exposure to PM2.5 has decreased in Russia over the years, resulting in a decrease in mortality associated with particulate matter air pollution. Throughout the analysis, mortality due to communicable diseases was low in both 1990 and 2019, while NCDs, such as ischemic heart disease and stroke, contributed to a significant number of deaths. These findings are consistent with other studies that suggest higher mortality from IHD, cerebrovascular diseases, and non-accidental causes due to higher concentrations of PM10 in the air of Moscow, Russia (Revich and Shaposhikov 2010). Similarly, research indicates that chronic cough and sputum production in the Murmansk region of northwest Russia are associated with outdoor air pollution, mostly high levels of sulfur dioxide (Nieminen et al. 2013).

The investigation into air pollution in India uncovers a startling phenomenon: a rise in PM2.5 air pollution exposure correlates with a decline in deaths linked to particulate matter pollution, notably from household sources. This unexpected trend is attributed to effective government policies, notably the Pradhan Mantri Ujjwala Yojana (PMUY), which advocates for cleaner cooking fuels such as LPG, thereby reducing reliance on solid fuels (Selvam et al. 2022; Singh and Dixit 2019). This shift has resulted in notable health improvements, particularly among women in rural households, such as reduced respiratory illnesses, eye problems, and burn injuries. The clean cooking fuel subsidy policy can improve rural health by reducing particulate matter pollution levels (Chakraborty and Mondal 2021; Dabadge et al. 2018). Overall, this research highlights the substantial impact of focused policy interventions in alleviating air pollution's health impacts and enhancing the welfare of Indian households.

In terms of exposure effects, China has similar results as Brazil and Russia when it comes to PM pollution deaths caused by PM2.5 exposure. Between 1990 and 2019, China saw relatively high deaths and disability-adjusted life years (DALYs) due to communicable diseases caused by particulate matter pollution, in comparison to non-communicable diseases (NCDs) for both genders. Health outcomes shifted distinctly as different types of air pollution took effect. In 2019, the health risks associated with ambient particulate matter air pollution surpassed those of household solid fuel air pollution in 1990, showcasing the unique lifestyle changes in China. More than half of the energy consumed by the Chinese population is derived from coal, taking into account China’s vast coal reserves. Additionally, rapid population growth and globalization have led to swift urbanization in China, resulting in increased use of products that emit significant amounts of air pollutants, such as private vehicles and household equipment. (Saini et al. 2021; Cropper 2010; Lin and Zhu 2018).

South Africa has a unique pattern of deaths related to PM2.5 air pollution. Despite a decrease in mean annual exposure to PM2.5 pollution, South Africa has experienced a significant increase in deaths associated with it. Consequently, South Africa is the only country with increasing death rates due to PM2.5 pollution. Health outcomes due to air pollution in South Africa are very similar to those in India. The paper's data shows a typical transition from a higher burden associated with communicable diseases to NCDs. Several factors make the South African population more vulnerable to air pollution, including housing conditions, co-morbidity, genetic background, socio-economic status, employment status, and education (Matooane et al. 2004). The study found that deaths and DALYs from ambient particulate matter air pollution increased, whereas household solid fuel air pollution tended to decline. Other studies have documented the significance of household air pollution in 1990 and following decades. For example, Barnes et al. found a positive association between lower respiratory infections due to indoor air pollution in children under 5 years (Barnes et al. (2009). Mortality risk from respiratory, cardiovascular, and cerebrovascular diseases due to ambient air pollution in South Africa was higher than in many developed countries, as suggested by Wichmann and Voyi (2005). Similarly, childhood asthma in South Africa is one of the biggest contributors to childhood DALYs and is associated with ambient air pollution (Olaniyan et al. 2015).

As five of the world's fastest-growing economies, the BRICS countries offer numerous opportunities for globalization, automation, urbanization, and population growth. However, they are also more susceptible to air pollution, which is a matter of concern. According to current statistics from GBD 2019, although the overall death rate and DALY rate have been decreasing since 1990 in all BRICS countries and globally, they still account for a significant proportion of all deaths and DALYs. This demands immediate policy interventions, especially in the post-pandemic Covid-19 context. While clinical advancement and modern public health approaches have helped prevent extreme health outcomes from communicable diseases, DALYs associated with NCDs, and even CDs, have led to more physical, mental, and economic losses.

This study, like others, exhibits certain limitations that warrant exploration in future research endeavors. While we have investigated the health impacts of air pollution with respect to gender and age, focusing on metrics such as mortality and disability-adjusted life years (DALYs), we have yet to delve into the effects across various communicable diseases (CDs) and non-communicable diseases (NCDs). Our study has primarily identified causes of deaths and DALYs attributed to environmental risk factors using a descriptive approach and data from the global burden of disease (GBD) survey. However, we have not empirically examined the direct effects of air pollution, such as CO2 emissions or PM2.5 annual exposure, on specific health outcomes like deaths and DALYs. Future research endeavors could delve into the intricate linkages between health outcomes, air pollution, and socio-economic factors through comprehensive time-series studies (Suthar et al. 2021). Moreover, this study exhibits limitations in terms of data quality, the potential for ecological fallacy, which may obscure individual-level variations, and oversight of spatial and temporal heterogeneity. Additionally, the study's precision in informing policy implications and generalizability may be constrained. Addressing these limitations in future research endeavors would contribute significantly to advancing our understanding of the complex interplay between air pollution and public health.

## Conclusion

The study conducted a comprehensive analysis of the impact of air pollution on public health in the BRICS nations over the period from 1990 to 2019. By examining data from the global burden of disease estimates, the research elucidated trends in deaths and disability adjusted life years (DALYs) attributable to air pollution, with a focus on disease patterns, age groups, and gender disparities.

The findings underscore a significant epidemiological transition, with communicable diseases being replaced by non-communicable diseases (NCDs) as the primary causes of air pollution-related deaths globally. This transition is particularly pronounced in China and India, where NCD-related deaths have surged over the study period. Moreover, the study highlights the vulnerability of specific age groups, including individuals aged 50–69 years, those aged 70 and above, and children under 5 years, to the adverse health effects of air pollution. Gender disparities are also evident, with males experiencing higher death rates and DALYs attributed to air pollution-related diseases compared to females.

In light of these findings, the study emphasizes the urgent need for evidence-based policymaking to address the escalating crisis of NCDs linked to air pollution. Targeted interventions must prioritize vulnerable age groups and address gender disparities to improve public health outcomes. By implementing effective strategies to mitigate air pollution's health impacts, policymakers can safeguard the health and well-being of populations in the BRICS nations and beyond

## Data Availability

Data is available in the public domain for research purposes and not for commercial use. Data can be obtained from the open-access repository of the Institute of Health Metrics Evaluation (IHME).

## Appendix

See Table 4, Figure 5, 6, 7.

**Table 4:** Death and DALYs rate (all ages) attributable to air pollution of communicable and non-communicable diseases in Global and BRICS (1990–2019)

Risk	Location	Communicable disease				Non-communicable disease			
		Death rate (per 100,000 population)		DALYs rate (per 100,000 population)		Death rate (per 100,000 population)		DALYs rate (per 100,000 population)	
		1990	2019	1990	2019	1990	2019	1990	2019
Air pollution	Global	40 (34–48)	15 (12–18)	3157 (2669–3759)	931 (761–1130)	81 (71–91)	72 (63–80)	2090 (1846–2334)	1825 (1611–2030)
	Brazil	16 (12–21)	4 (2–6)	1160 (873–1506)	144 (102–196)	26 (19–34)	24 (17–31)	1198 (869–1522)	643 (461–840)
	Russia	2 (1–4)	1 (1–2)	140 (96–201)	50 (30–79)	88 (42–136)	51 (30–74)	2056 (993–3172)	1154 (661–1655)
	India	69 (58–81)	20 (16–24)	5543 (4646–6508)	1170 (954–1431)	87 (73–101)	100 (84–117)	2505 (2155–2875)	2677 (2283–3087)
	China	26 (20–32)	4 (3–5)	1905 (1469–2351)	112 (86–141)	134 (112–157)	126 (107–148)	3240 (2726- 3787)	2877 (2453–3358)
	South Africa	34 (26–43)	13 (10–18)	2430 (1893–3117)	715 (519–947)	37 (31–42)	40 (33–48)	1125 (964–1281)	1109 (895–1317)
Ambient particulate matter pollution	Global	8 (5–12)	6 (4–8)	555 (320–909)	334 (237–445)	31 (22–40)	47 (39–55)	763 (551–1009)	1194 (980–1380)
	Brazil	4 (2–7)	3 (2–4)	260 (135–457)	99 (69–144)	1 (0–1)	17 (12–23)	466 (223–808)	465 (325–627)
	Russia	2 (1–3)	1 (1–2)	120 (73–182)	48 (28–77)	77 (33–126)	49 (28–71)	1810 (792–2957)	1102 (623–1596)
	India	13 (5–25)	11 (8–14)	1045 (431–1995)	641 (473–823)	20 (9–33)	59 (46–73)	560 (266–953)	1599 (1254–1950)
	China	6 (3–11)	3 (2–4)	438 (197–777)	83 (63–107)	38 (19–62)	97 (80–116)	916 (439–1498)	2227 (1828–2638)
	South Africa	14 (10–19)	11 (8–15)	955 (652–1299)	586 (412–777)	20 (15–25)	34 (26–41)	603 (463–748)	931 (729–1132)
Household pollution from solid fuels	Global	33 (25–42)	9 (6–11)	2602 (2001–3332)	597 (432–774)	49 (36–62)	21 (14–30)	1294 (980–1624)	585 (402–803)
	Brazil	12 (8–17)	1 (0–2)	900 (613–1245)	45 (22–76)	42 (31–54)	5 (2–10)	723 (533–944)	151 (70–269)
	Russia	0 (0–1)	0 (0–0)	20 (8–40)	2 (0–5)	9 (3–19)	2 (0–4)	205 (80–429)	37 (10–98)
	India	56 (41–71)	9 (6–12)	4498 (3284–5722)	529 (345–739)	66 (46–86)	35 (22–50)	1914 (1384–2468)	974 (645–1361)
	China	20 (13–27)	1 (0–1)	1467 (958–2014)	29 (15–48)	93 (63–124)	25 (12–42)	2253 (1540–2968)	586 (309–981)
	South Africa	20 (13–29)	2 (1–4)	1475 (953–2116)	129 (62–227)	16 (11–22)	6 (3–10)	515 (356–695)	162 (84–261)
Ambient ozone pollution	Global	No risk	No risk	No risk	No risk	4 (2–6)	5 (2–7)	74 (33–118)	80 (39–124)
	Brazil	No risk	No risk	No risk	No risk	16 (8–28)	2 (1–3)	11 (4–21)	29 (12–49)
	Russia	No risk	No risk	No risk	No risk	2 (1–4)	1 (0–2)	48 (20–78)	16 (6–28)
	India	No risk	No risk	No risk	No risk	5 (2–8)	12 (6–19)	108 (48–172)	220 (108–347)
	China	No risk	No risk	No risk	No risk	9 (4–14)	7 (3–11)	167 (73–270)	97 (44–156)
	South Africa	No risk	No risk	No risk	No risk	0 (0–1)	1 (0–2)	9 (4–17)	21 (9–35)

Note: Parenthesis denotes a 95% uncertainty interval of lower and upper limits. We have used 1990 as a reference period for our analysis. No risk = Communicable diseases have no risk for ambient ozone pollution as per the GBD data and it affects only the cause of non-communicable diseases which includes Chronic obstructive pulmonary disease. Source: Author’s estimation from the GBD-2019 Survey data (IHME 2020)

## Footnotes

1 All causes of death or DALYs rate means the number of deaths or DALYs due to all conditions (cause of disease). Cause means a single disease or injury or an aggregation of diseases and injuries that causes death or disability.
